# Māori Medical Student and Physician Exposure to Racism, Discrimination, Harassment, and Bullying

**DOI:** 10.1001/jamanetworkopen.2024.19373

**Published:** 2024-07-01

**Authors:** Donna Cormack, Claire Gooder, Rhiannon Jones, Cameron Lacey, James Stanley, Sarah-Jane Paine, Elana Curtis, Ricci Harris

**Affiliations:** 1 Te Kupenga Hauora Māori, Waipapa Taumata Rau, University of Auckland, Auckland, New Zealand; 2 Te Rōpū Rangahau Hauora a Eru Pomare (Eru Pōmare Māori Health Research Centre), Department of Public Health, University of Otago, Wellington, New Zealand; 3 Department of Psychological Medicine, University of Otago, Christchurch, New Zealand; 4 Department of Public Health, University of Otago, Wellington, New Zealand

## Abstract

**Importance:**

Discrimination, bullying, and harassment in medicine have been reported internationally, but exposures for Indigenous medical students and physicians, and for racism specifically, remain less examined.

**Objective:**

To examine the prevalence of racism, discrimination, bullying, and harassment for Māori medical students and physicians in New Zealand and associations with demographic and clinical characteristics.

**Design, Setting, and Participants:**

This cross-sectional study used data from an anonymous national survey of Māori medical students and physicians in New Zealand in late 2021 and early 2022. Data were analyzed from March 2022 to April 2024.

**Exposures:**

Age, gender, marginalized status (ie, in addition to being Māori, belonging to other groups traditionally marginalized or underrepresented in medicine), year of medical school, year of graduation, and main work role.

**Main Outcomes and Measures:**

Direct and witnessed racism, discrimination, bullying, and harassment were measured as any experience in the last year and ever. Any exposure to negative comments about social groups and witnessing discriminatory treatment toward Māori patients or whānau (extended family). Considering leaving medicine, including because of mistreatment, was measured.

**Results:**

Overall, 205 Māori medical students (median [IQR] age, 23.1 [21.6-24.3] years; 137 [67.2%] women) and 200 physicians (median [IQR] age, 36.6 [30.1-45.3] years; 123 [62.8%] women) responded. Direct and witnessed exposure to racism (184 students [91.5%]; 176 physicians [90.7%]) and discrimination (176 students [85.9%]; 179 physicians [89.5%]) ever in medical education, training, or work environments was common. Ever exposure to witnessed and direct bullying (123 students [66.5%]; 150 physicians [89.3%]) and harassment (73 students [39.5%]; 112 physicians [66.7%]) was also common. Most respondents reported witnessing Māori patients or their whānau being treated badly in clinical settings, in direct interactions (67 students [57.8%]; 112 physicians [58.9%]) or behind their backs (87 students [75.0%]; 138 physicians [72.6%]). One-quarter of Māori medical students (45 students), and 37.0% of physicians (61 physicians) had considered leaving or taken a break from medicine because of these experiences. Additional marginalized statuses were significantly associated with any direct experience of mistreatment in the last year for students and physicians. Exposure to some forms of mistreatment were also significantly associated with higher likelihood of thinking about leaving or taking a break from medicine for physicians.

**Conclusions and Relevance:**

In this study, Māori medical students and physicians reported high exposure to multiple forms of racism, discrimination, bullying, and harassment in medical education, training, and work environments, requiring an urgent response from medical institutions.

## Key Points

**Question:**

What is the prevalence of exposure to racism, discrimination, bullying, and harassment for Māori medical students and physicians in New Zealand and does it vary by medical student and physician characteristics?

**Findings:**

In this cross-sectional national survey of 405 Māori physicians and Māori medical students, direct and witnessed racism, discrimination, bullying, and harassment experiences were common, with some associations with gender, marginalized status, age for medical students and seniority for physicians.

**Meaning:**

These findings suggest that medical education and workplaces should address the high reported experience of multiple forms of racism, discrimination, bullying, and harassment for Māori medical students and physicians.

## Teaser

This cross-sectional study examines the prevalence of experiencing and witnessing racism, discrimination, harassment, and bullying (RDHB) for Māori medical students and physicians in New Zealand and associations with demographic and clinical characteristics.

## Introduction

Racism, discrimination, harassment, and bullying are increasingly acknowledged as substantial problems for medical students and physicians internationally,^1,2,3,4,5,6^ including in New Zealand.^7,8,9,10^ Research on these exposures only rarely examines experiences for Indigenous physicians and medical students specifically,^11,12,13,14^ despite calls for action by Indigenous peoples in medicine.^15,16,17^ Studies have not always examined racism separately from other forms of discrimination or assessed exposures by race or ethnicity.^18,19,20,21,22^

For Māori medical students and physicians in New Zealand, as for Indigenous medical professionals internationally, exposures to racism, discrimination, bullying, and harassment in medicine are intertwined with exposures to racism and other systems of oppression in society more generally. The critical Kaupapa Māori^23,24^ decolonial approach of this study recognizes that racism, discrimination, bullying, and harassment for Māori medical students and physicians happen within medical education and work contexts that are already racialized because of the primary structuring forces of colonialism and racism.^25,26,27^ Thus, how these are experienced for Māori students and physicians relative to other medical students and physicians may be qualitatively different.^27^

As part of the Te Whakahaumaru Taiao project on safe environments for Māori medical practitioners,^28^ national surveys were carried out in New Zealand to assess prevalence of racism, discrimination, bullying, and harassment in medical education, training, and workplaces and to explore impacts on health and careers for Māori medical students and physicians. This study reports findings on the prevalence of racism, other forms of discrimination, bullying, and harassment and associations of these with Māori medical student and physician characteristics.

## Methods

This cross-sectional study using survey data was approved by the Auckland Health Research Ethics Committee. Informed consent was provided by all participants electronically at the beginning of the survey. We followed the Strengthening the Reporting of Observational Studies in Epidemiology (STROBE) reporting guideline.

Data were collected via voluntary anonymous national-level online surveys (Qualtrics). All physicians and medical students identifying as Māori were invited to take part. Eligible participants were medical students enrolled in 2021 in years 2 through 6 at either of New Zealand’s 2 medical schools (University of Auckland and University of Otago) and all physicians with a current annual practicing certificate from the Medical Council of New Zealand. Further information on the New Zealand context is provided in eAppendix 1 in Supplement 1. Due to some inconsistency in reporting across the different institutions, we took the highest reported number of Māori medical students (491 students)^29^ and physicians (854 physicians) as response rate denominators to produce the most conservative estimate.

Invitations were sent to students by administrators and to physicians by Te Ohu Rata o Aotearoa (Māori Medical Practitioners Association), Medical Council of New Zealand, medical colleges, unions, academic institutions, and personal networks. The survey was also advertised on social media and the project website. Initial invitations were followed by reminder emails and additional advertising. The student survey had 2 waves (November 26 to December 24, 2021; February 24 to March 13, 2022) and the physician survey had 1 wave (January 20 to March 13, 2022). Participants could choose to receive a NZ$40 voucher (approximately US$25) for participation and/or go into a prize draw. Overall, 221 students and 210 physicians responded; however, 16 students and 10 physicians who did not complete any exposure questions were excluded.

### Measures

Survey development was informed by literature reviews, interviews with Māori medical students, and expert advisory input. As this was the first survey of its kind, it included both existing tools (some adapted) and new measures, reviewed by independent experts. Separate versions were prepared for medical students and physicians, with common questions and questions specific to each group (eAppendix 2 in Supplement 1).

#### Racism, Discrimination, Bullying, and Harassment

Participants were asked if they had directly experienced or witnessed (seen, heard, or heard about) another person or a group of people being subjected to discrimination, racism, bullying, or harassment, with definitions for each concept provided before the question (eAppendix 2 in Supplement 1). Students were asked about exposures during medical education and training, and physicians about exposures in the workplace and during work-related activities. Response options were “Yes, within the last 12 months”; “Yes, more than 12 months ago”; “No, never”; and “Don’t know.” Participants were able to select yes for both timeframes where applicable.

Experience of each of the 4 exposures (direct or witnessed) was recorded for the last 12 months. For analysis, an ever exposure category combined responses “Yes, within the last 12 months” and “Yes, more than 12 months ago.” We also created composite variables for any direct experience of racism, discrimination, bullying, or harassment (last 12 months, ever) and any witnessed racism, discrimination, bullying, or harassment (last 12 months, ever) and 8 variables for any direct or witnessed racism, discrimination, bullying, or harassment in the last 12 months and any direct or witnessed racism, discrimination, bullying, or harassment ever.

#### Stereotypes

We asked whether participants had seen or heard their colleagues or people in leadership roles make negative comments or jokes about several specific groups of people, including comments to or about patients, colleagues, or students. The groups of people asked about were those with a higher body mass index, lower socioeconomic status, of Māori ethnicity, of non-European ethnicity other than Māori (eg, Asian, Pacific), with lower levels of education, with disabilities, women, and/or people who are lesbian, gay, bisexual, transgender, queer, intersex, asexual, and others (LGBTQIA+); Rainbow; or Takatāpui. Response options were yes, no, and do not know.

#### Discriminatory Treatment

Students in the clinical years of their program (years 4-6) and all physicians were asked whether they had ever heard or seen Māori patients or whānau (ie, extended family) treated badly or treated less well than Pākehā (ie, individuals of European ethnicity) patients or family during direct interactions (ie, while patients or their whānau are present) or behind their backs (ie, while patients or their whānau are not present). Response options were yes, no, and do not know.

#### Leaving Medicine

Participants were asked if they had ever considered leaving medicine (Māori physicians) or ever considered dropping out of medicine (Māori medical students). Response categories were yes or no. A second question asked if they had ever considered taking a break from medicine because of racism, discrimination, bullying, or harassment in the workplace or training environment (physicians) or in the medical school or training environment (students). This could be the main reason or 1 factor. Response options were “Yes, I have considered it, but have not taken a break”; “Yes, I have considered it, and have taken a break”; “No, I have not considered it”; and “Don’t know.”

#### Demographics

For analysis, age (collected as month and year of birth to preserve anonymity) was calculated by assigning the 15th of the month as proxy birth date, then converting to age as of the date participants completed the survey. Age was analyzed in bands (students: <20-24, 25-29, ≥30 years; physicians: <30, 30-39, 40-49, 50-59, ≥60). Gender was asked as an open question (categorized as man, woman, nonbinary, and prefer not to say). To maintain anonymity given small numbers, nonbinary individuals were grouped with individuals who preferred not to provide their gender for analysis. To assess intersectional experiences, we also asked all participants to answer yes, no, or do not know to the question: “We have already asked you about being Māori. Apart from this identity, do you consider yourself to be a member of any other group that is traditionally underrepresented or marginalised in medicine?”

For analysis, students were further grouped into nonclinical (years 2-3) and clinical (years 4-6) training years. Physicians were asked about years since graduation (grouped into 4 categories for analysis). A composite category of junior (graduated in 2011 or later) and senior (graduated before 2011) status was also created. Physicians also reported their current main work role from the options of house officer, medical officer, registrar, specialist, general practitioner, or other (and they were asked to specify) (eTable in Supplement 1), with medical officer grouped with other for analysis, due to small numbers.

### Statistical Analysis

Data were analyzed in R statistical software version 4.2.1 (R Project for Statistical Computing). Frequencies, medians, IQRs, and 95% CIs were calculated. Two-sided χ^2^ tests were used to calculate *P* values, with significance set at *P* < .05. Data were analyzed from March 2022 to April 2024.

## Results

The final sample for analysis included 205 Māori medical students (response rate, 41.8%) and 200 Māori physicians (response rate, 23.4%). Median (IQR) age was 23.1 (21.6-24.3) years for Māori medical students, and 36.6 (30.1-45.3) years for Māori physicians (Table 1). By gender, 137 students (67.2%) and 123 physicians (62.8%) were women. In terms of marginalized status, 57 students (27.9%) and 59 physicians (29.5%) reported also belonging to other groups traditionally underrepresented or marginalized in medicine. Medical student respondents were relatively evenly distributed across study year (ranging from 19.0%-26.3%), except for a lower proportion (21 students [10.2%]) in sixth year. More than half the Māori physicians in our study had graduated in the last 12 years (106 physicians [53.8%]) and were categorized as junior for analysis. A total of 62 physicians (31.2%) indicated they were registrars and a further 56 physicians (28.1%) were specialists in their main current work role. A higher proportion of respondents in our sample were women, compared with medical students and physicians in New Zealand in general.^29,30,31^ Distribution by medical school was similar, but our study had a higher proportion of fourth-year students and a lower proportion of sixth-year students compared with all Māori medical students in 2021. Compared with the overall Māori medical workforce, the mean age of physicians in our study was similar (39.4 vs 39.0 years). Our respondents were more likely to be registrars and specialists as their main work role and less likely to be general practitioners, house officers, and medical officers.^31^

**Table 1.** Characteristics of Māori Medical Students and Physicians in Survey

Characteristic	Respondents, No./total No. (%)	
	Students (n = 205)	Physicians (n = 200)
Gender		
Woman	137/204 (67.2)	123/196 (62.8)
Man	61/204 (29.9)	68/196 (34.7)
Nonbinary or prefer not to say^a^	6/204 (2.9)	5/196 (2.6)
Age, median, (IQR), y	23.1 (21.6-24.3)	36.6 (30.1-45.3)
Marginalized status^b^		
Yes	57/204 (27.9)	59/200 (29.5)
No	135/204 (66.2)	138/200 (69.0)
Do not know	12/204 (5.9)	3/200 (1.5)
Year of medical school		
Second	39/205 (19.0)	NA
Third	44/205 (21.5)	NA
Fourth	54/205 (26.3)	NA
Fifth	47/205 (22.9)	NA
Sixth	21/205 (10.2)	NA
Year of graduation		
1990 and earlier	NA	22/197 (11.2)
1991-2000	NA	26/197 (13.2)
2001-2010	NA	43/197 (21.8)
2011-2020	NA	106/197 (53.8)
Main work role		
House officer	NA	31/199 (15.6)
Registrar	NA	62/199 (31.2)
Specialist	NA	56/199 (28.1)
General practitioner	NA	40/199 (20.1)
Other^c^	NA	10/199 (5.0)
Considered leaving medicine		
Yes	86/179 (48.0)	102/165 (61.8)
No	93/179 (52.0)	63/165 (38.2)
Considered taking a break because of racism, discrimination, bullying, and/or harassment		
Yes, but I did not	33/179 (18.4)	42/165 (25.5)
Yes, and I did	12/179 (6.7)	19/165 (11.5)
No, I have not considered it	126/179 (70.4)	98/165 (59.4)
Do not know	8/179 (4.5)	6/165 (3.6)

Abbreviation: NA, not applicable.

^a^ Nonbinary and prefer not to say categories were combined to reduce potential identifiability with small numbers.

^b^ Participants were asked, in addition to being Māori, if they identified with belonging to any other groups traditionally marginalized or underrepresented in medicine.

^c^ Includes medical officer and other.

Considering leaving medicine was common, reported by 102 Māori physicians (61.8%) and 86 Māori medical students (48.0%). One-quarter of students (45 students [25.1%] had considered taking a break, because of racism, discrimination, bullying, or harassment in medical school or training, including 33 students (18.4%) who had not taken a break and 12 students (6.7%) who had taken a break; 61 physicians (37.0%) considered taking a break because of racism, discrimination, bullying, or harassment in training or work environments, including 42 physicians (25.5%) who had not taken a break and 19 physicians (11.5%) who had taken a break.

Most Māori medical students had directly experienced (131 students [65.2%]) or witnessed (173 students [86.1%]) racism ever in their medical education (Table 2). For Māori physicians, 137 (70.6%) had experienced direct and 173 (89.2%) had witnessed racism in their workplaces or during work-related activities. Ever experiencing discrimination was also commonly reported for both students (119 students [58.3%] with direct experience and 169 students [82.4%] witnessed) and physicians (133 physicians [67.5%] with direct experience and 177 physicians [88.5%] witnessed).

**Table 2.** Frequency of Direct and Witnessed Discrimination, Racism, Bullying, and Harassment for Students and Physicians, by Time Period

Time period	Respondents, No./total No. (%)							
	Medical students				Physicians			
	Ever	Last year	Never	Do not know	Ever	Last year	Never	Do not know
Discrimination								
Direct	119/204 (58.3)	82/204 (40.2)	54/204 (26.5)	31/204 (15.2)	133/197 (67.5)	89/197 (45.2)	59/197 (30.0)	5/197 (2.5)
Witnessed	169/205 (82.4)	145/205 (70.7)	21/205 (10.2)	15/205 (7.3)	177/200 (88.5)	140/200 (70.0)	17/200 (8.5)	6/200 (3.0)
Any direct or witnessed	176/205 (85.9)	151/205 (73.7)	NR	NR	179/200 (89.5)	143/200 (71.5)	NR	NR
Racism								
Direct	131/201 (65.2)	92/201 (45.8)	55/201 (27.4)	15/201 (7.5)	137/194 (70.6)	91/194 (46.9)	51/194 (26.3)	6/194 (3.1)
Witnessed	173/201 (86.1)	150/201 (74.6)	15/201 (7.5)	13/201 (6.5)	173/194 (89.2)	138/194 (71.1)	18/194 (9.3)	3/194 (1.5)
Any direct or witnessed	184/201 (91.5)	158/201 (78.6)	NR	NR	176/194 (90.7)	141/194 (72.7)	NR	NR
Bullying^a^								
Direct	60/185 (32.4)	38/185 (20.5)	111/185 (60.0)	14/185 (7.6)	121/168 (72.0)	60/168 (35.7)	41/168 (24.4)	6/168 (3.6)
Witnessed	113/185 (61.1)	79/185 (42.7)	51/185 (27.6)	21/185 (11.4)	146/168 (86.9)	87/168 (51.8)	18/168 (10.7)	4/168 (2.4)
Any direct or witnessed	123/185 (66.5)	89/185 (48.1)	NR	NR	150/168 (89.3)	97/168 (57.7)	NR	NR
Harassment^a^								
Direct	42/185 (22.7)	25/185 (13.5)	130/185 (70.3)	13/185 (7.0)	80/168 (47.6)	35/168 (20.8)	76/168 (45.2)	12/168 (7.1)
Witnessed	65/184 (35.3)	40/184 (21.7)	91/184 (49.5)	28/184 (15.2)	96/165 (58.2)	48/165 (29.1)	48/165 (29.1)	21/165 (12.7)
Any direct or witnessed	73/185 (39.5)	50/185 (27.0)	NR	NR	112/168 (66.7)	60/168 (35.7)	NR	NR
Any racism, discrimination, bullying, or harassment^a^								
Direct	171/205 (83.4)	137/205 (66.8)	NR	NR	174/200 (87.0)	126/200 (63.0)	NR	NR
Witnessed	196/205 (95.6)	182/205 (88.8)	NR	NR	193/200 (96.5)	164/200 (82.0)	NR	NR

Abbreviation: NR, not reported to avoid overreporting at the composite level.

^a^ The questions about bullying and harassment were asked later in the survey and have a lower denominator as some people had dropped out of the survey by this point.

Ever exposure to witnessed and direct bullying (123 students [66.5%]; 150 physicians [89.3%]) was common. Overall, 121 Māori physicians (72.0%) had directly experienced and 146 Māori physicians (86.9%) had witnessed bullying, higher than for Māori medical students, with 60 students (32.4%) reporting direct experience and 113 students (61.1%) reporting having witnessed bullying. Harassment, while lower, was still common, with 73 students (39.5%) and 112 physicians (66.7%) reporting ever exposure. Harassment was experienced directly by 42 medical students (22.7%) and 80 physicians (47.6%) and witnessed by 65 students (35.3%) and 96 physicians (58.2%). Recent experiences (last 12 months) are reported in Table 2. In the last year, most Māori medical students and physicians had experienced directly (137 students [66.8%]; 126 physicians [63.0%]) or witnessed (182 students [88.8%]; 164 physicians [82.0%]) at least 1 form of racism, discrimination, bullying, or harassment. Māori medical students and physicians reported directly experiencing racism (92 students [45.8%]; 91 physicians [46.9%]), discrimination (82 students [40.2%]; 89 physicians [45.2%]), bullying (38 students [20.5%]; 60 physicians [35.7%]), and harassment (25 students [13.5%]; 35 physicians [20.8%]) in the last year.

Most participants had heard negative comments or jokes in medical education, training, or work environments about people with higher weight or BMI (152 students [76.8%]; 164 physicians [88.2%]), about Māori (117 students [59.4%]; 131 physicians [70.1%]) and other non-European (120 students [60.6%]; 146 physicians [77.7%]) ethnic groups, women (117 students [59.1%]; 103 physicians [54.8%]), people with lower socioeconomic status (93 students [47.0%]; 122 physicians [64.9%]), or people with perceived lower levels of education (100 students [50.8%]; 108 physicians [57.4%]) (Table 3). Hearing jokes or negative comments about people with disabilities, while lower, was still reported by 49 medical students (24.9%) and 49 physicians (26.1%) (Table 3). Among 116 students and 190 physicians who responded to the question, reports for witnessing racism toward Māori patients and their whānau while patients or whānau were present and while they were not present were similar for students in their clinical years (67 students [57.8%] witnessed racism when patients or whānau were present; 87 students [75.0%] witnessed racism when patients or whānau were not present) and physicians (112 physicians [58.9%] witnessed racism when patients or whānau were present; 138 physicians [72.6%] when patients or whānau were not present) (Table 3).

**Table 3.** Exposures to Stereotypes and Witnessed Racism Toward Māori Among Māori Medical Students and Māori Physicians

Exposure	Students			Physicians		
	Yes	No	Do not know	Yes	No	Do not know
Stereotypes						
People who have a higher BMI	152/198 (76.8)	34/198 (17.2)	12/198 (6.1)	164/186 (88.2)	22/186 (11.8)	0
People who have lower socioeconomic status	93/198 (47.0)	89/198 (45.0)	16/198 (8.1)	122/188 (64.9)	63/188 (33.5)	3/188 (1.6)
People who are Māori	117/197 (59.4)	65/197 (33.0)	15/197 (7.6)	131/187 (70.1)	51/187 (27.3)	5/187 (2.7)
People who are non-European, other than Māori (eg, Asian, Pacific)	120/198 (60.6)	60/198 (30.3)	18/198 (9.1)	146/188 (77.7)	38/188 (20.2)	4/188 (2.1)
People who are LGBTQIA+/Rainbow/Takatāpui	84/198 (42.4)	95/198 (48.0)	19/198 (9.6)	84/188 (44.7)	95/188 (50.5)	9/188 (4.8)
People with lower levels of education	100/197 (50.8)	76/197 (38.6)	21/197 (10.7)	108/188 (57.4)	67/188 (35.6)	13/188 (6.9)
People with disabilities	49 /197 (24.9)	129/197 (65.5)	19/197 (9.6)	49/188 (26.1)	126/188 (67.0)	13/188 (6.9)
Women	117/198 (59.1)	68/198 (34.3)	13/198 (6.6)	103/188 (54.8)	80/188 (42.6)	5/188 (2.7)
Racism toward Māori patients or whānau^a^						
During direct interactions (ie, while patient/whānau are present)	67/116 (57.8)	37/116 (31.9)	12/116 (10.3)	112/190 (58.9)	70/190 (36.8)	8/190 (4.2)
Behind their backs (ie, while patient or whānau are not present)	87/116 (75.0)	24/116 (20.7)	5/116 (4.3)	138/190 (72.6)	49/190 (25.8)	3/190 (1.6)

Abbreviations: BMI, body mass index; LGBTQIA+, lesbian, gay, bisexual, transgender, queer, intersex, asexual, and others.

^a^ The questions about racism toward Māori patients or whānau questions were only asked of students in the clinical years (years 4-6).

Women reported higher prevalence of discrimination, racism, bullying (physicians only), and harassment (physicians only) for any direct or witnessed mistreatment in the last year compared with men (Table 4). This difference was statistically significant for any racism for medical students (86.8% [95% CI, 80.0%-91.5%] of women vs 58.3% [95% CI, 45.7%-69.9%] of men) (Figure). Except for harassment, exposure was higher in the older age groups for students, with a statistically significant association for bullying (age <25 years, 44.0% [95% CI, 36.0%-52.2%] vs age ≥30 years, 84.6% [95% CI, 57.8%-95.7%]). In contrast, younger physicians generally reported the highest exposures, with the exception of harassment. Having at least 1 other marginalized status was associated with higher likelihood of experiencing any discrimination (86.0% [95% CI, 74.7%-92.7%]) and racism (87.3% [95% CI, 76.0%-93.7%]) for students and any direct form of racism, discrimination, bullying, or harassment for both students (80.7% [95% CI, 68.7%-88.9%]) and physicians (76.3% [95% CI, 64.0%-85.3%]).

**Table 4.** Racism, Discrimination, Bullying and Harassment in Last Year by Characteristics of Māori Medical Students and Physicians

Characteristic	Respondents, No./total No. (%) [95% CI]					
	Any discrimination in last year	Any racism in last year	Any bullying in last year	Any harassment in last year	Any direct RDBH in last year	Any witnessed RDBH in last year
**Students**						
Gender						
Woman	105/137 (76.6) [68.9-82.9]	118/136 (86.8) [80.0-91.5]	60/127 (47.2) [38.8-55.9]	30/127 (23.6) [17.1-31.7]	96/137 (70.1) [61.9-77.1]	124/137 (90.5) [84.4-94.4]
Man	39/61 (63.9) [51.4-74.8]	35/60 (58.3) [45.7-69.9]	28/54 (51.9) [38.9-64.6]	17/54 (31.5) [20.7-44.7]	35/61 (57.4) [44.9-69.0]	52/61 (85.2) [74.3-92.0]
Nonbinary or prefer not to say^a^	6/6 (100) [61.0-100]	5/5 (100) [56.6-100]	1/4 (25.0) [4.6-69.9]	3/4 (75.0) [30.1-95.4]	5/6 (83.3) [43.6-97.0]	5/6 (83.3) [43.6-97.0]
*P* value	.06	<.001	.55	.05	.15	.51
Age, y						
<25	111/155 (71.6) [64.1-78.1]	120/154 (77.9) [70.7-83.7]	62/141 (44.0) [36.0-52.2]	41/141 (29.1) [22.2-37.0]	103/155 (66.5) [58.7-73.4]	136/155 (87.7) [81.6-92.0]
25-29	23/31 (74.2) [56.8-86.3]	22/30 (73.3) [55.6-85.8]	15/28 (53.6) [35.8-70.5]	6/28 (21.4) [10.2-39.5]	20/31 (64.5) [46.9-78.9]	28/31 (90.3) [75.1-96.7]
≥30	13/15 (86.7) [62.1-96.3]	13/14 (92.9) [68.5-98.7]	11/13 (84.6) [57.8-95.7]	2/13 (15.4) [4.3-42.2]	12/15 (80.0) [54.8-93.0]	14/15 (93.3) [70.2-98.8]
*P* value	.45	.33	.02	.44	.53	.77
Other marginalized status^b^						
Yes	49/57 (86.0) [74.7-92.7]	48/55 (87.3) [76.0-93.7]	26/51 (51.0) [37.7-64.1]	13/51 (25.5) [15.5-38.9]	46/57 (80.7) [68.7-88.9]	53/57 (93.0) [83.3-97.2]
No	93/135 (68.9) [60.6-76.1]	104/135 (77.0) [69.3-83.3]	57/123 (46.3) [37.8-55.1]	33/123 (26.8) [19.8-35.3]	82/135 (60.7) [52.3-68.6]	119/135 (88.1) [81.6-92.6]
Do not know	8/12 (66.7) [39.1-86.2]	6/11 (54.5) [28.0-78.7]	6/11 (54.5) [28.0-78.7]	4/11 (36.4) [15.2-64.6]	8/12 (66.7) [39.1-86.2]	9/12 (75.0) [46.8-91.1]
*P* value	.04	.04	.78	.76	.03	.19
Clinical years						
Nonclinical years	57/83 (68.7) [58.1-77.6]	61/82 (74.4) [64.0-82.6]	36/77 (46.8) [36.0-57.8]	18/77 (23.4) [15.3-34.0]	50/83 (60.2) [49.5-70.1]	71/83 (85.5) [76.4-91.5]
Clinical years	94/122 (77.0) [68.8-83.6]	97/119 (81.5) [73.6-87.5]	53/108 (49.1) [39.8-58.4]	32/108 (29.6) [21.8-38.8]	87/122 (71.3) [62.7-78.6]	111/122 (91.0) [84.6-94.9]
*P* value	.24	.30	.24	.44	.13	.32
Considered leaving medicine						
Yes	70/86 (81.4) [71.9-88.2]	72/86 (83.7) [74.5-90.0]	42/86 (48.8) [38.6-59.2]	28/86 (32.6) [23.6-43.0]	68/86 (79.1) [69.3-86.3]	79/86 (91.9) [84.1-96.0]
No	64/93 (68.8) [58.8-77.3]	71/93 (76.3) [66.8-83.8]	45/93 (48.4) [38.5-58.4]	22/93 (23.7) [16.2-33.2]	58/93 (62.4) [52.2-71.5]	83/93 (89.2) [81.3-94.1]
*P* value	.08	.30	>.99	.25	.02	.73
Considered leaving medicine because of RDBH						
Yes, but I did not	27/33 (81.8) [65.6-91.4]	28/33 (84.8) [69.1-93.3]	22/33 (66.7) [49.6-80.2]	14/33 (42.4) [27.2-59.2]	29/33 (87.9) [72.7-95.2]	31/33 (93.9) [80.4-98.3]
Yes, and I did	10/12 (83.3) [55.2-95.3]	9/12 (75.0) [46.8-91.1]	5/12 (41.7) [19.3-68.0]	4/12 (33.3) [13.8-60.9]	7/12 (58.3) [32.0-80.7]	11/12 (91.7) [64.6-98.5]
No, I have not considered it	90/126 (71.4) [63.0-78.6]	99/126 (78.6) [70.6-84.8]	57/126 (45.2) [36.8-53.9]	29/126 (23.0) [16.5-31.1]	84/126 (66.7) [58.1-74.3]	113/126 (89.7) [83.1-93.9]
Do not know	7/8 (87.5) [52.9-97.8]	7/8 (87.5) [52.9-97.8]	3/8 (37.5) [13.7-69.4]	3/8 (37.5) [13.7-69.4]	6/8 (75.0) [40.9-92.9]	7/8 (87.5) [52.9-97.8]
*P* value	.43	.78	.14	.14	.09	.88
**Physicians**						
Gender						
Woman	93/123 (75.6) [67.3-82.3]	92 /119 (77.3) [69.0-83.9]	62/103 (60.2) [50.5-69.1]	38/103 (36.9) [28.2-46.5]	84/123 (68.3) [59.6-75.9]	104/123 (84.6) [77.1-89.9]
Man	45/68 (66.2) [54.3-76.3]	44/66 (66.7) [54.7-76.8]	31/57 (54.4) [41.6-66.6]	19/57 (33.3) [22.5-46.3]	36/68 (52.9) [41.2-64.3]	53/68 (77.9) [66.7-86.2]
Nonbinary or prefer not to say^a^	2/5 (40.0) [11.8-76.9]	2/5 (40.0) [11.8-76.9]	2/4 (50.0) [15.0-85.0]	3/4 (75.0) [30.1-95.4]	3/5 (60.0) [23.1-88.2]	4/5 (80.0) [37.6-96.4]
*P* value	.11	.08	.74	.25	.11	.52
Age, y						
<30	33/40 (82.5) [68.1-91.3]	34/38 (89.5) [75.9-95.8]	25/35 (71.4) [54.9-83.7]	15/35 (42.9) [28.0-59.1]	28/40 (70.0) [54.6-81.9]	38/40 (95.0) [83.5-98.6]
30-39	55/77 (71.4) [60.5-80.3]	57/75 (76.0) [65.2-84.2]	37/66 (56.1) [44.1-67.4]	18/66 (27.3) [18.0-39.0]	46/77 (59.7) [48.6-70.0]	63/77 (81.8) [71.8-88.8]
40-49	29/42 (69.0) [54.0-80.9]	25/42 (59.5) [44.5-73.0]	17/35 (48.6) [33.0-64.4]	13/35 (37.1) [23.2-53.7]	28/42 (66.7) [51.6-79.0]	30/42 (71.4) [56.4-82.8]
50-59	17/24 (70.8) [50.8-85.1]	17/24 (70.8) [50.8-85.1]	12/21 (57.1) [36.5-75.5]	9/21 (42.9) [24.5-63.5]	16/24 (66.7) [46.7-82.0]	20/24 (83.3) [64.1-93.3]
≥60	8/15 (53.3) [30.1-75.2]	7/13 (53.8) [29.1-76.8]	6/10 (60.0) [31.3-83.2]	4/10 (40.0) [16.8-68.7]	7/15 (46.7) [24.8-69.9]	11/15 (73.3) [48.0-89.1]
*P* value	.29	.02	.41	.49	.51	.08
Other marginalized status						
Yes	46/59 (78.0) [65.9-86.6]	45/56 (80.4) [68.2-88.7]	32/48 (66.7) [52.5-78.3]	19/48 (39.6) [27.0-53.7]	45/59 (76.3) [64.0-85.3]	50/59 (84.7) [73.5-91.8]
No	96/138 (69.6) [61.4-76.6]	95/135 (70.4) [62.2-77.4]	64/117 (54.7) [45.7-63.4]	41/117 (35.0) [27.0-44.0]	80/138 (58.0) [49.6-65.9]	113/138 (81.9) [74.6-87.4]
Do not know	1/3 (33.3) [6.1-79.2]	1/3 (33.3) [6.1-79.2]	1/3 (33.3) [6.1-79.2]	0	1/3 (33.3) [6.1-79.2]	1/3 (33.3) [6.1-79.2]
*P* value	.16	.11	.25	.37	.03	.08
Year of graduation						
1990 and earlier	12/22 (54.5) [34.7-73.1]	12/20 (60.0) [38.7-78.1]	8/15 (53.3) [30.1-75.2]	4/15 (26.7) [10.9-52.0]	10/22 (45.5) [26.9-65.3]	16/22 (72.7) [51.8-86.8]
1991-2000	20/26 (76.9) [57.9-89.0]	19/26 (73.1) [53.9-86.3]	13/21 (61.9) [40.9-79.2]	12/21 (57.1) [36.5-75.5]	17/26 (65.4) [46.2-80.6]	20/26 (76.9) [57.9-89.0]
2001-2010	31/43 (72.1) [57.3-83.3]	27/43 (62.8) [47.9-75.6]	15/39 (38.5) [24.9-54.1]	10/39 (25.6) [14.6-41.1]	27/43 (62.8) [47.9-75.6]	33/43 (76.7) [62.3-86.8]
2011-2020	79/106 (74.5) [65.5-81.9]	82/102 (80.4) [71.6-86.9]	60/91 (65.9) [55.7-74.8]	33/91 (36.3) [27.1-46.5]	70/106 (66.0) [56.6-74.4]	93/106 (87.7) [80.1-92.7]
*P* value	.26	.08	.03	.09	.34	.18
Junior and senior status						
Junior (graduated 2011-2020)	79/106 (74.5) [65.5-81.9]	82/102 (80.4) [71.6-86.9]	60/91 (65.9) [55.7-74.8]	33/91 (36.3) [27.1-46.5]	70/106 (66.0) [56.6-74.4]	93/106 (87.7) [80.1-92.7]
Senior (graduated 2010 or earlier)	63/91 (69.2) [59.1-77.8]	58/89 (65.2) [54.8-74.3]	36/75 (48.0) [37.1-59.1]	26/75 (34.7) [24.9-45.9]	54/91 (59.3) [49.1-68.9]	69/91 (75.8) [66.1-83.5]
*P* value	.50	.03	.03	.96	.41	.05
Main work role						
House officer	25/31 (80.6) [63.7-90.8]	27/29 (93.1) [78.0-98.1]	19/23 (82.6) [62.9-93.0]	10/23 (43.5) [25.6-63.2]	21/31 (67.7) [50.1-81.4]	30/31 (96.8) [83.8-99.4]
Registrar	46/62 (74.2) [62.1-83.4]	47/60 (78.3) [66.4-86.9]	33/54 (61.1) [47.8-73.0]	21/54 (38.9) [27.0-52.2]	43/62 (69.4) [57.0-79.4]	52/62 (83.9) [72.8-91.0]
Specialist	38/56 (67.9) [54.8-78.6]	33/55 (60.0) [46.8-71.9]	25/48 (52.1) [38.3-65.5]	18/48 (37.5) [25.2-51.6]	33/56 (58.9) [45.9-70.8]	43/56 (76.8) [64.2-85.9]
General practitioner	24/40 (60.0) [44.6-73.7]	26/40 (65.0) [49.5-77.9]	14/33 (42.4) [27.2-59.2]	7/33 (21.2) [10.7-37.8]	21/40 (52.5) [37.5-67.1]	29/40 (72.5) [57.2-83.9]
Other^c^	9/10 (90.0) [59.6-98.2]	7/9 (77.8) [45.3-93.7]	5/9 (55.6) [26.7-81.1]	4/9 (44.4) [18.9-73.3]	8/10 (80.0) [49.0-94.3]	9/10 (90.0) [59.6-98.2]
*P* value	.19	.01	.04	.38	.30	.07
Considered leaving medicine						
Yes	73/102 (71.6) [62.2-79.4]	77/102 (75.5) [66.3-82.8]	66/102 (64.7) [55.1-73.3]	41/102 (40.2) [31.2-49.9]	73/102 (71.6) [62.2-79.4]	89/102 (87.3) [79.4-92.4]
No	46/63 (73.0) [61.0-82.4]	42/63 (66.7) [54.4-77.1]	27/62 (43.5) [31.9-55.9]	16/62 (25.8) [16.6-37.9]	35/63 (55.6) [43.3-67.2]	49/63 (77.8) [66.1-86.3]
*P* value	.98	.29	.01	.09	.05	.17
Considered leaving medicine because of RDBH						
Yes, but I did not	35/42 (83.3) [69.4-91.7]	36/42 (85.7) [72.2-93.3]	28/42 (66.7) [51.6-79.0]	17/42 (40.5) [27.0-55.5]	35/42 (83.3) [69.4-91.7]	38/42 (90.5) [77.9-96.2]
Yes, and I did	16/19 (84.2) [62.4-94.5]	18/19 (94.7) [75.4-99.1]	15/19 (78.9) [56.7-91.5]	11/19 (57.9) [36.3-76.9]	17/19 (89.5) [68.6-97.1]	18/19 (94.7) [75.4-99.1]
No, I have not considered it	64/98 (65.3) [55.5-74.0]	61/98 (62.2) [52.4-71.2]	46/97 (47.4) [37.8-57.3]	26/97 (26.8) [19.0-36.4]	52/98 (53.1) [43.3-62.6]	77/98 (78.6) [69.5-85.5]
Do not know	4/6 (66.7) [30.0-90.3]	4/6 (66.7) [30.0-90.3]	4/6 (66.7) [30.0-90.3]	3/6 (50.0) [18.8-81.2]	4/6 (66.7) [30.0-90.3]	5/6 (83.3) [43.6-97.0]
*P* value	.10	.004	.03	.04	<.001	.17

Abbreviation: RDBH, racism, discrimination, bullying, or harassment.

^a^ Nonbinary and prefer not to say categories were combined to reduce potential identifiability with small numbers.

^b^ Participants were asked, in addition to being Māori, if they identified with belonging to any other groups traditionally marginalized or underrepresented in medicine.

^c^ Includes medical officer and other.

**Figure.**
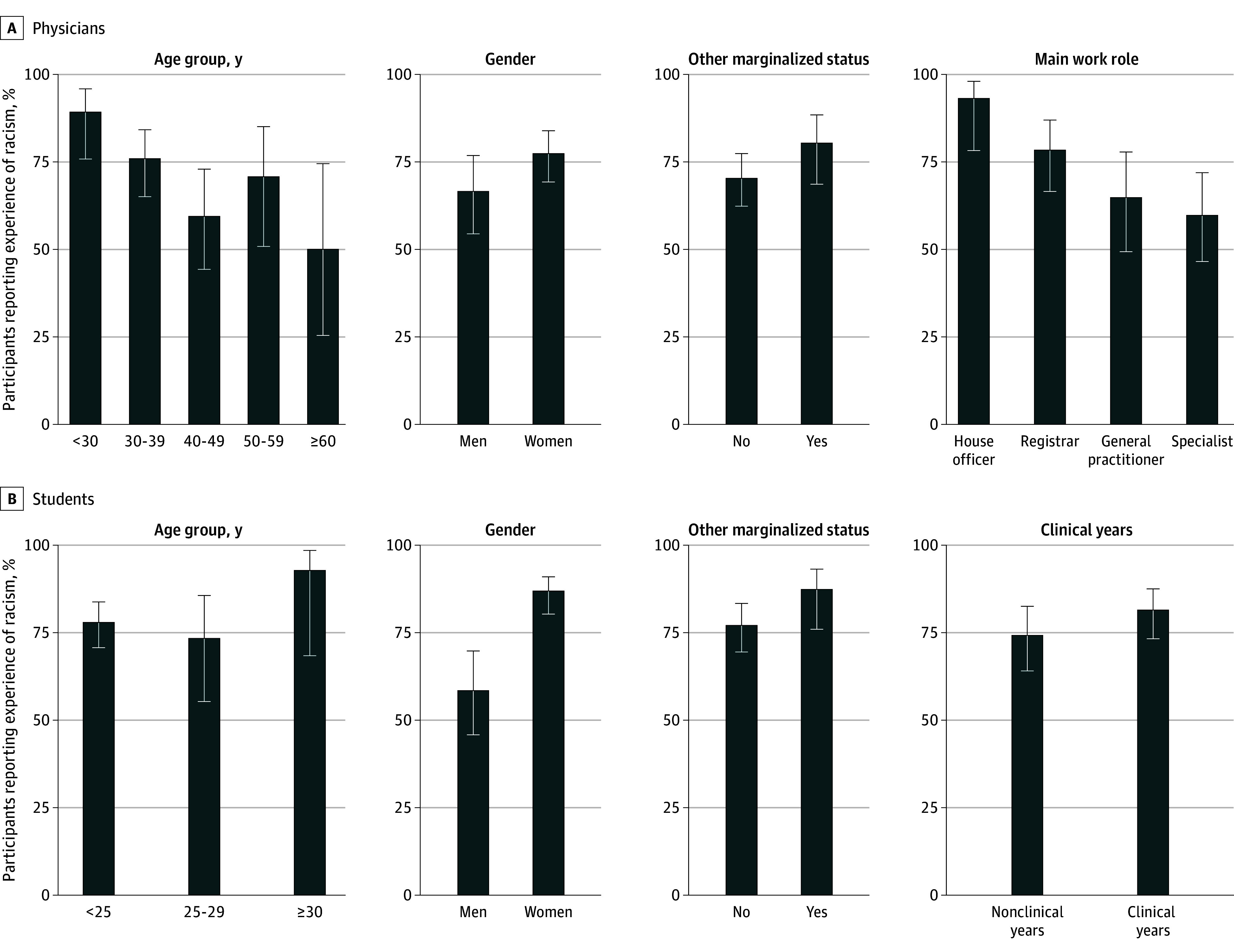
Racism in the Last Year by Characteristics of Māori Medical Students and Physicians

Physicians who had graduated after 2011 reported higher exposure to any racism (80.4% [95% CI, 71.6%-86.9%] vs 65.2% [95% CI, 54.8%-74.3%]), any witnessed mistreatment (87.7% [95% CI, 80.1%-92.7%] vs 75.8% [95% CI, 66.1%-83.5%]), any bullying (65.9% [95% CI, 55.7%-74.8%]) vs 48.0% [95% CI, 37.1%-59.1%]) than physicians who had graduated 10 or more years ago. House officers also reported the highest exposure to racism, direct and witnessed (93.1% [95% CI, 78.0%-98.1%]). Finally, experiencing any direct mistreatment was associated with higher report of considering leaving medicine for medical students (79.1% [95% CI, 69.3%-86.3%] vs 62.4% [95% CI, 52.2%-71.5%]), and experiencing bullying with higher report of considering leaving medicine for physicians (64.7% [95% CI, 55.1%-73.3%] vs 43.5% [95% CI, 31.9%-55.9%]). For Māori physicians, experiencing racism, bullying, harassment, and any direct forms of mistreatment were also statistically significantly associated with higher report of considering leaving medicine because of discrimination, racism, bullying, and harassment (Table 4).

## Discussion

This cross-sectional study using data from a national survey of Māori medical students and physicians identified high levels of exposure to racism, other discrimination, bullying, and harassment, supporting reports by Māori medical students and Māori physicians over many years.^32,33,34^ Racism was experienced directly or witnessed by almost all Māori medical students and physicians in our study. In contrast to other studies, we specifically asked questions about racism separate from discrimination, in line with our theoretical approach that conceptualizes racism as a primary experience for Indigenous peoples and an organizing feature of exposure to oppressive systems, harmful environments, and mistreatment at an individual level. The high prevalence of exposure to racism for both time periods examined, and across the continuum of medical education, training, and work, highlights the persistent, ubiquitous nature of racism for Indigenous medical students and physicians. This aligns with a recent Australian study that reported high prevalence of racism for Aboriginal and Torres Strait Islander medical trainees, with 20% directly experiencing and 30% witnessing racism in the last 12 months, higher than the national response in the same survey (6% and 13%, respectively).^14^

Prevalence of discrimination, bullying, and harassment ever and in the last year was also consistently high for Māori physicians in our study and higher than other studies of Australian and New Zealand physicians. For example, Māori physicians in our study reported direct exposure to discrimination (45.2%), bullying (35.7%), and harassment (20.8%) in the last year. In comparison, a 2016 study of Australasian intensive care medicine fellows and trainees reported a prevalence of 32% for bullying and 12% for discrimination in the 12 months prior.^21^ A recent study of senior medical officers in New Zealand reported 37.2% experienced bullying in the last 6 months.^35^ The finding that Māori medical students and physicians report not only high exposure to racism, but also the same or higher prevalence of other forms of discrimination is consistent with research in New Zealand on multiple forms of discrimination.^36^

Witnessed experiences also can have an impact on medical students and physicians and contribute to harmful learning and working environments. Students and physicians reported hearing negative comments and jokes about groups of people related to weight, ethnicity, gender, and sexuality, and perceived socioeconomic characteristics. Stereotypes about people with disabilities were reported at relatively lower frequencies by both Māori physicians and students. This may reflect actual lower prevalence or the normalization of ableist language in medicine meaning lower reporting.^37,38^ Exposure to these stereotypes is concerning in terms of the safety of Māori medical students and physicians who are members of these groups, as well as potential impacts on patients and patient care. In addition, most physicians and clinical-year students in this study reported witnessing Māori patients and/or their whānau treated badly or worse than New Zealand European people either directly or behind their backs.

High proportions of Māori medical students (48%) and physicians (62%) had considered leaving medicine in our study. While there are not directly comparable measures, this is higher than the 18% of senior physicians and dentists in New Zealand who reported they intended to leave in the next 5 years.^39^ Importantly, one-quarter of Māori medical students and more than one-third of physicians had thought about or taken a break from medicine specifically because of racism, bullying, discrimination, or harassment. This is likely to be an underestimate, as those students or physicians currently on a break or who have left medicine would not have been eligible to take part in our survey. A recent survey with British medical students and physicians found that 23% of physicians had considered leaving and 9% had left medicine because of racial discrimination,^40^ and research in the US has shown that physicians who experience workplace discrimination are more likely to consider leaving medicine.^41^

The impact of racism, discrimination, bullying, and harassment on workforce retention is an underrecognized and underaddressed issue in terms of current health care workforce shortages. However, it is important to highlight that regardless of whether these exposures impacted negatively on medical students, physicians, or on patients, they are unacceptable. Māori medical students and physicians have a right to learn, train, and work in environments that are free from oppression.

A strength of this study is its wider focus in assessing direct and witnessed experiences, and the broader environment. It likely still understates these exposures, as we asked about experiences ever and in the last 12 months, but not the frequency of these experiences; therefore we did not capture multiple exposures. However, we did include a separate module focused on measuring experiences of everyday racism, including perpetrators and settings, which will be reported in a separate publication. A further strength is the specific focus on Māori, taking into account the particular racialized, colonial contexts for Māori medical students and physicians. Our estimated response rates indicate that the survey included more than 40% of all Māori medical students and approximately one-quarter of all Māori physicians in 2021. Other studies in this area have generally not separated information out for Māori (or Indigenous) medical students and physicians or grouped all New Zealand respondents together with Australian respondents. In addition, the national nature of this survey that includes both medical students and physicians means it captured the experiences across the continuum of medicine.

### Limitations

This study has some limitations. As a survey, there may be selection bias in those who chose to participate. This could plausibly include a stronger propensity to respond among those with more experience of the exposures studied or who identify more strongly as Māori. While response rates were reasonably high by modern standards, the reported results should be considered as upper bounds for the prevalence of these exposures. Additionally, in interpreting the study findings, we recognize that the COVID-19 pandemic may have impacted some of the responses to questions about the last 12 months or for people who had joined medical school during the pandemic (eg, more online teaching, less clinical exposure), and that this may have changed the patterning of responses for some of the exposures.

## Conclusions

This cross-sectional study found that Māori medical students and physicians are not currently able to train and work in safe environments. These findings require an urgent and systematic response from medical schools and workplaces to ensure that medicine is safe for Indigenous medical students, physicians, and communities.

## Notes

Supplement 1. **eAppendix 1.** New Zealand Medical School, Training and Work Context **eAppendix 2.** Further Detail on Survey Measures **eTable.** Explanation of Work Roles

Supplement 2.
Data Sharing Statement
