# Supplementary material for: Māori Medical Student and Physician Exposure to Racism, Discrimination, Harassment, and Bullying
Source: JAMA Netw Open. 2024 Jul 1;7(7):e2419373. doi: 10.1001/jamanetworkopen.2024.19373 (PMC11217868; doi:10.1001/jamanetworkopen.2024.19373)
Supplement: Supplement 1. — eAppendix 1. New Zealand Medical School, Training and Work Context eAppendix 2. Further Detail on Survey Measures eTable. Explanation of Work Roles [file jamanetwopen-e2419373-s001.pdf]

# Supplemental Online Content

Cormack D, Gooder C, Jones R, et al. Māori medical student and physician exposure to racism, discrimination, harassment, and bullying. *JAMA Netw Open*. 2024;7(7):e2419373. doi:10.1001/jamanetworkopen.2024.19373

**eAppendix 1.** New Zealand Medical School, Training and Work Context
**eAppendix 2.** Further Detail on Survey Measures
**eTable.** Explanation of Work Roles

This supplemental material has been provided by the authors to give readers additional information about their work.

© 2024 Cormack D et al. *JAMA Network Open.*

**eAppendix 1: New Zealand medical school, training and work context**

New Zealand's (NZ's) medical training is a 6-year undergraduate programme, offered at two universities (the University of Otago and the University of Auckland). The first year is a common health sciences or biomedical sciences year, with selection into medicine at the end of that year. There is also a graduate entry admission pathway for students who already have an undergraduate degree. Years 2 and 3 are primarily non-clinical, university-based training. Years 4 to Years 6 are primarily clinical, hospital or community-based training. The final year (6$^{th}$ year) is also referred to as a Trainee Intern year.

Following the final year of medical school, a hospital-based internship is completed. To practise medicine in NZ, doctors need to hold a current practicing certificate. This is applied for annually and is referred to as an Annual Practising Certificate (APC). [1] The general term in NZ is doctors, rather than physician. Physician is a more specialised term that applies only to some doctors.

Doctors also can choose to specialise by undergoing additional specialist training.[1] The length of this training depends on the speciality. Many specialist medical colleges in NZ are joint colleges with Australia.

© 2024 Cormack D et al. *JAMA Network Open.*

**eAppendix 2: Further detail on survey measures**
This Appendix includes some further context and detail on variables that are included in this paper.

***Māori identity and descent***
Eligibility to participate in this survey was limited to medical students and doctors who self-identified as Māori, based on ethnicity, descent or both. In NZ, ethnicity is understood as a measure of cultural affiliation with the ethnic group or groups that a person identifies with. To assess ethnicity in this survey, we used the official statistical question from the NZ Census of Population and Dwellings, which asks "Which ethnic group do you belong to? Mark the space or spaces which apply to you".[2] Eight standard response options, including Māori, are provided, as well as a free text option. We included anyone who reported Māori as their only, or one of their, ethnicities.

We also asked a descent question which asked "Do you whakapapa Māori? For example, do you have a Māori parent, grandparent or great-grandparent?". This was adapted from a question from the New Zealand Health Survey and the Te Kupenga Māori Social Survey. Anyone who responded yes to this question was also eligible to be included in the survey.

***Discrimination***
Discrimination was measured with the following two questions, one assessing direct experiences and one assessing witnessed experiences. Students were asked these questions about medical education and training, and doctors were asked about workplace exposures. The definition of discrimination is adapted from two pieces of NZ legislation, *Human Rights Act, 1993* and *Employment Relations Act, 2000.*
<u>Direct discrimination</u>
During your medical education or training/In the workplace and/or during work-related activities have you ever *experienced* discrimination? *<u>Discrimination</u> is when a person is treated unfairly or less favourably because of a particular characteristic or status (e.g. religion, ethnicity, marital status) than another person in the same or similar circumstances. This can result in differential access to conditions, benefits, and opportunities. You can select both 'yes' options if applicable.*
Response options: Yes, within the last 12 months; Yes, more than 12 months ago; No, never; Don't know
<u>Witnessed discrimination</u>
During your medical education or training/In the workplace and/or during work-related activities have you ever *witnessed* (seen, heard or heard about) another person or a group of people being subjected to discrimination?
Response options: Yes, within the last 12 months; Yes, more than 12 months ago; No, never; Don't know

***Racism***
Racism was measured with the following two questions, one assessing direct experiences and one assessing witnessed experiences. Students were asked these questions about medical education and training, and doctors were asked about workplace exposures. The definition is drawn from health and racism literature.[3-5]
<u>Direct racism</u>
During your medical education or training/In the workplace and/or during work-related activities have you ever *experienced* racism? *Racism is a system that perpetuates the power and privilege held by one ethnic/racial group by oppressing and devaluing other ethnic/racial groups, using the invented concepts of 'race' and racial hierarchies. Colonisation forms the main context for understanding how racism manifests in Aotearoa. Racism occurs at all levels - from interpersonal to societal. It can involve things like racially motivated hate crimes, jokes or stereotypes, racial slurs, or unequal treatment. You can select both 'yes' options if applicable.*
Response options: Yes, within the last 12 months; Yes, more than 12 months ago; No, never; Don't know
<u>Witnessed racism</u>
During your medical education or training/In the workplace and/or during work-related activities have you ever *witnessed* (seen, heard or heard about) another person or a group of people being subjected to racism? *You can select both 'yes' options if applicable.*
Response options: Yes, within the last 12 months; Yes, more than 12 months ago; No, never; Don't know

***Bullying***
Bullying was measured with the following two questions, one assessing direct experiences and one assessing witnessed experiences. Students were asked these questions about medical education and training, and doctors were asked about workplace exposures. For respondents who said they had experienced bullying in the last 12 months, follow up questions about the types of bullying experienced were asked, but are not reported in this paper. The definition for bullying was adapted from a NZ medical student survey,[6] and the New Zealand Nursing Organisation (NZNO) policy on workplace bullying.[7]
<u>Direct bullying</u>

© 2024 Cormack D et al. *JAMA Network Open.*

During your medical education or training/In the workplace and/or during work-related activities have you ever *experienced* bullying? *Bullying is behaviour that a person finds offensive, intimidating, abusive or humiliating so as to have a detrimental effect upon a person's dignity, safety, and well-being. Bullying is intentional and can be overt or covert, and can be perpetrated by anyone in any position in a training or workplace setting. You can select both 'yes' options if applicable.*
Response options: Yes, within the last 12 months; Yes, more than 12 months ago; No, never; Don't know
Witnessed bullying
During your medical education or training/In the workplace and/or during work-related activities have you ever *witnessed* (seen, heard, heard about) another person or a group of people being subjected to bullying? *You can select both 'yes' options if applicable.*
Response options: Yes, within the last 12 months; Yes, more than 12 months ago; No, never; Don't know

***Harassment***
Harassment was measured with the following two questions, one assessing direct experiences and one assessing witnessed experiences. Students were asked these questions about medical education and training, and doctors were asked about workplace exposures. For respondents who said they had experienced harassment in the last 12 months, follow up questions about the types of non-sexual harassment experienced were asked, but are not reported in this paper. The definitions were drawn from the *Harassment Act, 1997* and a NZ medical student survey.[6]
Direct harassment
During your medical education or training/In the workplace and/or during work-related activities have you ever *experienced* harassment? *Harassment is an unwanted pattern of behaviour that seeks to intimidate or pressure you either directly or indirectly. This includes non-sexual and/or sexual harassment.* Such conduct creates an intimidating, hostile or offensive environment. *You can select both 'yes' options if applicable.*
Response options: Yes, within the last 12 months; Yes, more than 12 months ago; No, never; Don't know
Witnessed harassment
During your medical education or training/In the workplace and/or during work-related activities have you ever *witnessed* (seen, heard, heard about) another person or a group of people being subjected to harassment? *You can select both 'yes' options if applicable.*
Response options: Yes, within the last 12 months; Yes, more than 12 months ago; No, never; Don't know

***Stereotypes***
A question was asked about witnessing stereotypes in medical education, training and work settings. The question was influenced by a question in the CHANGES Study.[8] Our adapted question asked:
In your medical education or training/the workplace and/or during work-related activities have you seen or heard your colleagues and/or people in leadership roles make negative comments or jokes about the following groups of people: *This includes comments to or about patients, colleagues or students.*
The groups of people asked about were: People who have a higher BMI; People who have lower socioeconomic status; People who are Māori; People who are non-European, other than Māori (e.g., Pacific, Asian); People who are LGBTQIA+/Rainbow/Takatāpui; People with lower levels of education; People with disabilities; and, Women.
Response options: Yes; No; Don't know

***Witnessing racism towards Māori patients and/or their whānau***
All doctors and medical students in their clinical years (3-6) were asked about hearing or seeing racism towards Māori patients and/or their whānau. The question asked:
In medical education or training/In the workplace and/or during work-related activities have you ever heard or seen Māori patients/whānau treated badly or treated less well than Pākehā patients/family – either in direct interactions with them (e.g. bedside, during whānau discussion) or while they are not present (e.g. before walking into a room, while debriefing)?
The two items were: During direct interactions (i.e. while patient/whānau are present); Behind their backs (i.e. while patient/whānau are not present).
Response options were: Yes, No, Don't know

© 2024 Cormack D et al. *JAMA Network Open.*

***Leaving medicine***

Two questions were asked about leaving medicine, a general question and one that was specifically related to exposure to racism, discrimination, bullying or harassment. The general question was based on one from a survey with NZ tertiary students,[9] the specific question was new.

Leaving medicine

Have you ever considered dropping out of medical school? [students] / Have you ever considered leaving medicine? [doctors]

Response options: Yes; No

Leaving medicine because of mistreatment

Have you ever considered taking a break from medicine because of racism, discrimination, bullying and/or harassment in the medical school or training environment? *This could be the main reason or one factor.* [students]

Have you ever considered taking a break from medicine because of racism, discrimination, bullying and/or harassment in the workplace or training environment? *This could be the main reason or one factor* [doctors]

Response options: Yes, I have considered it, but have not taken a break; Yes, I have considered it, and have taken a break; No, I have not considered it; Don't know

***Marginalised identities***

In order to assess intersectionality, we asked a question of all participants on their identification with groups that are marginalised in medicine, outside of their identity as Māori. The wording of this questions was:

*We have already asked you about being Māori. Apart from this identity, do you consider yourself to be a member of any other group that is traditionally underrepresented or marginalised in medicine?*

Response options: Yes; No; Don’t know

© 2024 Cormack D et al. *JAMA Network Open.*

**eTable 1: Explanation of work roles**

**House officer:** This refers to a junior doctor who is usually in their first two years out of medical school. House officers are also sometimes referred to as interns, house surgeons, or by their post-graduate year (e.g. PGY1).[10]

**General practitioner:** A doctor who is working in the role of a general practitioner (or primary care doctor), including doctors who have a fellowship from the Royal New Zealand College of General Practitioners or general practice as a vocational scope.[10]

**Medical Officer:** A medical officer refers to a doctor who is not training or has not specialised yet, or may not meet the eligibility requirements in New Zealand for a specialist or consultant role.[11]

**Registrar:** A registrar is also referred to as a trainee and is generally undertaking specialist training in a vocational training programme (MCNZ). They have at least two years of experience since medical school.[10]

**Specialist:** A specialist, also referred to as a consultant, is a senior doctor who has specialised in a particular area of medicine and is a member of their specialist college.[10]

© 2024 Cormack D et al. *JAMA Network Open.*

**References:**


1. Medical Council New Zealand (MCNZ). Medical education and training. Accessed April 5, 2024. https://www.mcnz.org.nz/registration/medical-education/
2. Ministry of Health.  *HISO 10001:2017 Ethnicity Data Protocols.* Ministry of Health (NZ); 2017..
3. Talamaivao N, Harris R, Cormack D, Paine SJ, King P. Racism and health in Aotearoa New Zealand: a systematic review of quantitative studies. *NZ Med J*. 2020;133(1521):55-68.
4. Okechukwu CA, Souza K, Davis KD, Butch de Castro A. Discrimination, harassment, abuse, and bullying in the workplace: Contribution of workplace injustice to occupational health disparities. *Am J Ind Med*. 2014;57(5):573-586.
5. Pihama L. Colonization and the importation of ideologies of race, gender, and class in Aotearoa. In: McKinley E, Tuhiwai Smith L, eds. *Handbook of Indigenous education*. Springer Nature; 2017:1–20.
6. New Zealand Medical Students' Association (NZMSA). Clinical student survey: Bullying and sexual harassment experience in clinical placements of 4, 5, 6th year medical students.2015.
7. New Zealand Nurses Organisation (NZNO). Workplace bullying policy. Accessed April 5 2024. https://www.nzno.org.nz/bullyfree
8. Burke SE, Dovidio JF, Perry SP, Burgess DJ, Hardeman RR, Phelan SM, Cunningham BA, Yeazel MW, Przedworski JM, van Ryn M. Informal training experiences and explicit bias against African Americans among medical students. *Soc Psychol Q*. 2017;80(1):65-84. doi: 10.1177/0190272516668166
9. New Zealand Union of Students' Associations (NZUSA). Kei Te Pai? Report on student mental health in Aotearoa. 2018.
10. Medical Council of New Zealand (MCNZ)*The New Zealand medical workforce in 2022*. Medical Council of New Zealand; 2022.
11. Medical Council of New Zealand (MCNZ)*Glossary*. Accessed April 5, 2024. https://www.mcnz.org.nz/about-us/glossary/


© 2024 Cormack D et al. *JAMA Network Open.*
