# Supplementary material for: Māori Medical Student and Physician Exposure to Racism, Discrimination, Harassment, and Bullying
Source: JAMA Netw Open. 2024 Jul 1;7(7):e2419373. doi: 10.1001/jamanetworkopen.2024.19373 (PMC11217868; doi:10.1001/jamanetworkopen.2024.19373)
Supplement: Supplement 2. — Data Sharing Statement [file jamanetwopen-e2419373-s002.pdf]

Data Sharing Statement

Cormack. Māori Medical Student and Physician Exposure to Racism, Discrimination, Harassment, and Bullying. *JAMA Netw Open*. Published July 01, 2024. doi:10.1001/jamanetworkopen.2024.19373

Data
Data available: No

Additional Information
Explanation for why data not available: As part of our ethics approval, we are not able to make individual-level data available. We can provide a data dictionary on request, and numbers are provided in the manuscript.
